# Improvement of hip abductor muscle weakness after lumbar decompressive surgery

**DOI:** 10.3109/03009734.2012.708678

**Published:** 2012-10-30

**Authors:** Tatsuro Sasaji, Kiyoshi Horaguchi, Noboru Yamada, Kazuo Iwai

**Affiliations:** Department of Orthopedic Surgery, Fukushima Rosai Hospital, 3-Numajiri, Tsuzura-machi, Uchigo, Iwaki 973-8403, Japan

**Keywords:** Decompressive surgery, degenerative lumbar spinal disorder, hip abductor muscle, L5 radiculopathy, muscle weakness

## Abstract

**Introduction.:**

Degenerative lumbar spinal disorder is common in Japan, and the L5 nerve root is commonly involved in this disorder. The symptoms of L5 radiculopathy are irradiating lateral leg pain, and numbness and weakness of tibialis anterior and the hip abductor muscle. There has been only one report on the results of surgery for hip abductor muscle weakness caused by degenerative lumbar spinal disorder.

**Patients and methods.:**

In this study, we analyzed the strength of the hip abductor muscle before and after decompressive surgery in 26 cases and the relationship between the lumbar disc herniation (LDH) and lumbar spinal canal stenosis (LSCS) groups.

**Results.:**

Of the total 26 cases, muscle strength improved in 23 cases (88%), with complete recovery in 17 cases (65%). In the LDH group, the improvement rate was 92%. In the LSCS group, the improvement rate was 68%. Although the improvement rate for the LDH group was higher than that for the LSCS group, the difference was not significant (*P* = 0.054).

**Discussion.:**

Decompressive surgery may be an effective method to improve hip abductor muscle weakness in degenerative lumbar spinal disorder.

## Introduction

Japan is an aging society, and the incidence of degenerative lumbar spinal disorder is increasing. The major neurological symptoms of lumbar spinal disorder are radicular pain and numbness and sometimes muscle weakness in the leg. Improvement of leg muscle strength is critical because it interferes with daily life. L5 radiculopathy causes muscle weakness not only in tibialis anterior (TA), but also in the hip abductor muscle (1). Surgical results for drop foot have been reported (2-5). However, there was only one report of surgical results on hip abductor muscle weakness (4). In this study, we report surgical results on hip abductor muscle weakness from degenerative lumbar spinal disorder.

## Patients and methods

All patients were informed that the data from their cases would be used for this study.

### Subjects

This study included 26 consecutive patients (10 females and 16 males, aged 24–82 years, mean age 66 years) who were referred to our institute for surgical treatment between 2010 and 2011 and underwent follow-up examinations for a minimum of 1 year. The mean follow-up period was 14 months (range 12–22 months). The diagnosis of degenerative lumbar spinal disorder was based on clinical symptoms, neurological examinations, and magnetic resonance imaging of the lumbar spine. All patients had typical neurological symptoms of L5 radiculopathy including irradiating lateral leg pain and numbness, and weakness of hip abductor muscle. The degenerative lumbar spinal disorders included lumbar disc herniation (LDH) and lumbar spinal canal stenosis (LSCS). The LSCS cases included central canal stenosis and foraminal stenosis. In LDH and central canal stenosis, the L4–5 segment was involved. In foraminal stenosis, the L5–S segment was involved. The exclusion criteria for this study included prior lumbar spinal surgery and osteoarthritis of the hip. The LDH group included 6 males and 5 females with a mean ± SD age at the time of surgery of 56 ± 18 years (range 24–77 years). The LSCS group included 10 males and 5 females with a mean ± SD age at the time of surgery of 72 ± 7 years (range 60–83 years) (Table I). The mean age was significantly higher in the LSCS group than in the LDH group (*P* = 0.02, Student's *t* test). This study did not include unstable spinal cases, and thus decompressive surgery was performed without fusion.

**Table I.** Improvement of hip abductor muscle strength.

	LDH (*n* = 11)	LSCS (*n* = 15)
Age, years	56 ± 18	72 ± 7
Mean preoperative muscle strength, arbitrary units, 0–5	2.7	2.6
Mean postoperative muscle strength, arbitrary units, 0–5	4.8	4.3
Improvement rate (%)	92	68

The mean age between groups at the time of surgery was significantly different (*P* = 0.02). The improvement rates between LDH and LSCS groups were not significantly different (*P* = 0.054). LDH = lumbar disc herniation; LSCS = lumbar spinal canal stenosis.

### Surgical procedures

**Figure 1.**
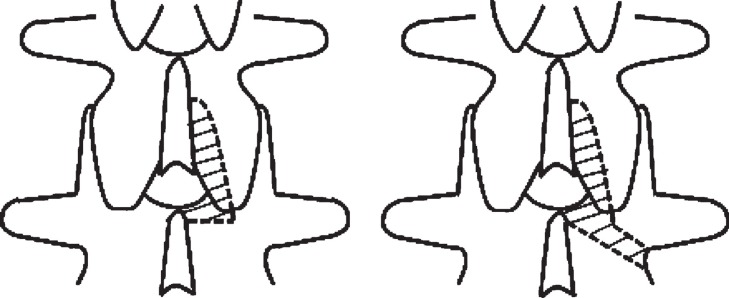
Schemata of surgeries. These schemata show decompression of the right side. The shaded area is the decompressed area. The wide fenestration decompressed the dura and nerve root in the entry zone (left). The wide fenestration and lateral fenestration decompressed the dura and nerve root in the entry, mid, and exit zones (right).

All surgeries were performed by a single spinal surgeon. For LDH, a simple open discectomy was performed. For central canal stenosis, wide fenestration was performed (Figure 1) (6). For foraminal stenosis, wide fenestration and lateral fenestration were performed (Figure 1).

### Clinical evaluation

We evaluated the strength of the hip abductor muscle before surgery and at the final follow-up examination using a manual muscle test. Muscle strength was graded on a 0 (paralysis) to 5 (normal strength) (arbitrary units) scale (Table II) (7).

**Table II.** Muscle strength (7).

Grade, arbitrary units	Characteristic
5	Active movement against full resistance
4	Active movement against gravity and some resistance
3	Active movement against gravity
2	Active movement with gravity eliminated
1	Trace movement or barely detectable contraction
0	No muscular contraction identified

### Calculation of percentage of improvement rate

The relationship between the LDH and LSCS groups and the improvement rates in hip abductor muscle weakness after surgery were analyzed. Surgical results were measured by postoperative muscle strength. The percentage of improvement rate was calculated as follows: (postoperative muscle strength − preoperative muscle strength)/(5 – preoperative muscle strength) ×100.

### Statistical analysis

For statistical purposes, the improvement rate data were compared using the Student's *t*-test. A probability value of less than 0.05 was considered significant. Statistical analyses were performed using GraphPad Prism (GraphPad Software, Inc., San Diego, CA, USA).

## Results

**Figure 2.**
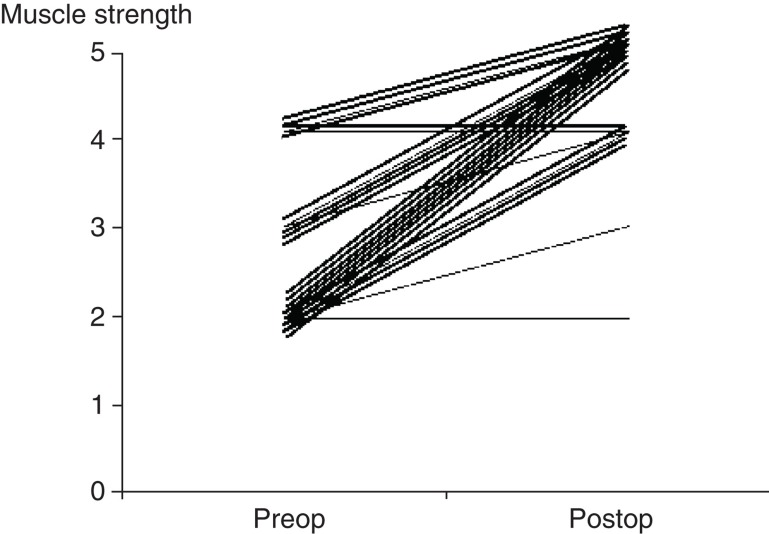
Changes in muscle strength. Twenty-three cases improved postoperatively including seventeen cases with a complete recovery. Muscle strength improved from 2 to 5 in eight cases, from 3 to 5 in five cases, from 4 to 5 in four cases, from 2 to 4 in four cases, from 3 to 4 in one case, and from 2 to 3 in one case. Preop = preoperative. Postop = postoperative.

There were 11 LDH cases and 15 LSCS cases. Six patients had preoperative muscle strength of 4, six patients had preoperative muscle strength of 3, and fourteen patients had preoperative muscle strength of 2. At the final follow-up examination, seventeen patients had postoperative muscle strength of 5, seven patients had muscle strength of 4, one patient had a muscle strength of 3, and one patient had a muscle strength of 2 (Figure 2). Of the 26 patients, 23 patients showed postoperative improvement and 3 patients showed no change. The mean improvement rate was 78%, and full recovery was achieved in 17 cases (65%). All of the unchanged cases were LSCS and males with a mean age of 78 years (range 71–83 years).

In the LDH group, the mean preoperative muscle strength was 2.7 (range 2–4), and the mean postoperative muscle strength was 4.8 (range 4–5). The improvement rate was 92%. In the LSCS group, the mean preoperative muscle strength was 2.6 (range 2–4), and the mean postoperative muscle strength was 4.3 (range 2–5). The improvement rate was 68%. Although the improvement rate was higher in the LDH group, this increase was not significant (*P* = 0.054) (Table I).

## Discussion

The L5 nerve root is commonly involved in degenerative lumbar spinal disorder (1). The L5 nerve root innervates extensor hallucis longus, tibialis anterior, and the hip abductor muscle (1). There has been only one report on surgical outcomes for hip abductor muscle weakness (4). According to Lee et al., in case of unilateral hip abductor muscle weakness caused by L5 radiculopathy, the pelvis of the affected side inclined upward and the pelvis of the unaffected side inclined downward (8). If postoperative improvement of hip abductor muscle strength is inadequate, the gait disturbance would be expected to continue. Therefore, we concluded that it was necessary to investigate the improvement rate in hip abductor muscle strength after surgery.

In the study by Girardi et al., 94% of patients experienced full recovery of abductor strength, but the strength of hip abductor muscle was not reported (4). In this study, the full recovery rate was 65%. We could not compare the full recovery rate.

In this study, the improvement rate in hip abductor muscle strength did not differ significantly between the LDH and LSCS groups despite the higher mean age in the LSCS group. We assumed that hip abductor muscle weakness caused by either LSCS or LDH would be indicative of the need for decompressive surgery. However, the number of cases in this study was small. Therefore, further studies with a larger number of patients would be necessary to address this question.
